# On tests of treatment-covariate interactions: An illustration of appropriate power and sample size calculations

**DOI:** 10.1371/journal.pone.0177682

**Published:** 2017-05-17

**Authors:** Gwowen Shieh

**Affiliations:** Department of Management Science, National Chiao Tung University, Hsinchu, Taiwan; University of New South Wales, AUSTRALIA

## Abstract

The appraisals of treatment-covariate interaction have theoretical and substantial implications in all scientific fields. Methodologically, the detection of interaction between categorical treatment levels and continuous covariate variables is analogous to the homogeneity of regression slopes test in the context of ANCOVA. A fundamental assumption of ANCOVA is that the regression slopes associating the response variable with the covariate variable are presumed constant across treatment groups. The validity of homogeneous regression slopes accordingly is the most essential concern in traditional ANCOVA and inevitably determines the practical usefulness of research findings. In view of the limited results in current literature, this article aims to present power and sample size procedures for tests of heterogeneity between two regression slopes with particular emphasis on the stochastic feature of covariate variables. Theoretical implications and numerical investigations are presented to explicate the utility and advantage for accommodating covariate properties. The exact approach has the distinct feature of accommodating the full distributional properties of normal covariates whereas the simplified approximate methods only utilize the partial information of covariate variances. According to the overall accuracy and robustness, the exact approach is recommended over the approximate methods as a reliable tool in practical applications. The suggested power and sample size calculations can be implemented with the supplemental SAS and R programs.

## Introduction

The existence of interactive phenomena between predictor variables on the response variable is an essential issue in all scientific studies. The detection of interactions between categorical treatment levels and continuous covariate variables is equivalent to the test of homogeneity of regression slopes test in ANCOVA designs. Notably, ANCOVA represents a constructive synthesis of analysis of variance and multiple linear regression to account for the relationship between the response variable and the concomitant or covariate variables in treatment comparisons. In addition to the fundamental assumptions of independence, normality, and constant variance, the within-group regression coefficients of the criterion variable on the covariate variable are presumed to be equal in ANCOVA. Violation of the ANCOVA assumptions has been the target of attention in the literature such as Glass, Peckham, and Sanders [1] and Harwell [2]. Naturally, the actual significance level and power of the regular test for treatment effects can be distorted to some extent under nonparallel regression settings. Hence, the validity of heterogeneity regression slopes plays a crucial role in applying the traditional ANCOVA or generalized alternatives. As a general guideline, a test for nonparallel regression lines is required as the preliminary procedure for use of traditional ANCOVA. If the test for heterogeneity of regression slopes is significant, then it suggests that the standard ANCOVA is no longer an appropriate technique. Accordingly, Fleiss [3], Huitema [4], and Maxwell and Delaney [5] provide comprehensive exposition and general strategy under heterogeneity of regression.

The statistical perspectives and appropriate strategies of covariate selection are presented in Hauck, Anderson, and Marcus [6], Hernandez, Steyerberg, and Habbema [7], Pocock et al. [8], Raab, Day, and Sales [9], and references therein. Moreover, the impact of omitted covariates on the statistical inferences has been demonstrated in Hauck et al. [10], Gail, Wieand, and Plantadosi [11], and Negassa and Hanley [12]. However, there is no related exploration about the direct consequence of excluding covariate characteristics in power and sample size calculations. In view of the potential applicability in practice, this article focuses on the most fundamental ANCOVA designs for two treatment groups and a single covariate. For the purposes of planning research designs and validating crucial interactions, power and sample size procedures were considered in Dupont and Plummer [13]. Their formula is very attractive from a computational standpoint and has been implemented in statistical packages. However, it is important to note that the particular method involves several convenient approximations including the use of a shifted *t* distribution for a noncentral *t* distribution and the substitution of fixed parameters for random covariates. The inherent nature and implications of accuracy were not addressed in Dupont and Plummer [13]. Accordingly, the existing illustrations were not detailed enough to elucidate the potential deficiency of their approximate technique. Because of the limited results in the literature, the current article aims to contribute to the development of power and sample size methodology for the tests for heterogeneity of two regression slopes. The emphasis is placed on the practical situation that not only the values of response variables for each subject are just available after the observations are made, but also the levels of covariate variables cannot be predetermined before data collection.

It is noteworthy that a different and prominent situation of interactive research involves interactions between two continuous covariates. Although the model formulations and test procedures of the interactive analysis are rather similar for the two types of covariate variable combination: continuous by continuous and categorical by continuous, their test statistics and associated distribution properties are considerably different. Therefore, the power and sample size calculations of Shieh [14] for detecting interactions between two continuous variables in multiple regression settings are not appropriate for assessing interactions between grouping and continuous variables within the context of ANCOVA. In a continual effort to support the analytical development and improve the essence of research findings in interaction studies, this investigation updates and expands the previous work of Dupont and Plummer [13] in such a way that the findings not only notify the fundamental deficiency of existing procedure, but also reinforce the usefulness of interaction designs in applications.

The present study has three key aspects. First, to account for the stochastic nature of covariate variables, the covariates are assumed to follow a normal distribution. Both exact and approximate power functions and sample size procedures for detecting heterogeneity of regression slopes are derived. Second, extensive numerical examinations were conducted to examine the deficiency of the approximate methods and the advantage of the exact approach under a wide range of model settings. The performance and robustness of the described techniques with respect to non-normality of the covariates are also investigated. Third, in view of the limited features of existing software packages, both SAS [15] and R [16] computer algorithms are developed to facilitate the implementation of the suggested power and sample size computations.

## Methods

The two-group nonparallel simple linear regression model is of the form

$$Y_{1j}=\beta_{01}+X_{1j}\beta_{11}+\varepsilon_{1j}\;\text{and}\;Y_{2k}=\beta_{02}+X_{2k}\beta_{12}+\varepsilon_{2k},$$ (1)

where *ε*_1*j*_ and *ε*_2*k*_ are *iid N*(0,*σ*^2^) random variables, *j* = 1,…, *N*_1_, and *k* = 1,…, *N*_2_. It is often informative to rewrite the regression model with heterogeneous slopes in Eq 1 as the following interactive multiple regression model using a dummy variable *M*:

$$Y_{i}=\beta_{02}+M_{i}\beta_{0D}+X_{i}\beta_{12}+M_{i}X_{i}\beta_{1D}+\varepsilon_{i},i=1,\ldots ,N,N=N_{1}+N_{2},$$ (2)

$$where\;\beta_{0D}=\beta_{01}-\beta_{02},\beta_{1D}=\beta_{11}-\beta_{12};$$

$$Y_{i}=Y_{1j},X_{i}=X_{1j},\varepsilon_{i}=\varepsilon_{1j},\;and\;M_{i}=1\;if\;i=j,j=1,\ldots ,N_{1};$$

$$Y_{i}=Y_{2k},X_{i}=X_{2k},\varepsilon_{i}=\varepsilon_{2k},\;and\;M_{i}=0\;if\;i=N_{1}+k,k=1,\ldots ,N_{2}.$$

Note that a traditional ANCOVA model assumes that the regression slopes are equivalent *β*_11_ = *β*_12_ = *β*_1_ and it postulates the parallel regression formulation

$$Y_{i}=\beta_{02}+M_{i}\beta_{0D}+X_{i}\beta_{1}+\varepsilon_{i},i=1,\ldots ,N.$$ (3)

Because the strategy and procedure for treatment comparisons differ for the nonparallel and parallel regression frameworks, the equality of covariate regression coefficients is viewed as the most crucial assumption in ANCOVA. Accordingly, a test for heterogeneity of regression slopes is generally required to justify the use of ANCOVA. When the assumption of equal within-group covariate regression coefficients is not tenable, the standard procedures of ANCOVA are no longer appropriate and alternative methods such as Johnson-Neyman and Picked-Point solutions for heterogeneous regression should be adopted. More conceptual and thorough discussions of alternative solutions to traditional ANCOVA can be found in Rogosa [17] and Rutherford [18].

In order to facilitate the detection of heterogeneous regression slopes, this article describes and examines the corresponding procedures for power and sample size determinations. Under the heterogeneous linear model assumption defined in Eq 1, it follows from standard results that the least squares estimators ${\hat{\beta }}_{11}$ and ${\hat{\beta }}_{12}$ of slope coefficients *β*_11_ and *β*_12_ have the following distributions

$${\hat{\beta }}_{11}∼N(\beta_{11},\sigma^{2}/{SSX}_{1})\;\mathrm{and}\;{\hat{\beta }}_{12}∼N(\beta_{12},\sigma^{2}/{SSX}_{2}),$$

where ${SSX}_{1}=\sum_{j=1}^{N_{1}}{\left(X_{1j}-{\bar{X}}_{1}\right)}^{2}$ and ${SSX}_{2}=\sum_{k=1}^{N_{2}}{\left(X_{2k}-{\bar{X}}_{2}\right)}^{2}$, ${\bar{X}}_{1}$ and ${\bar{X}}_{2}$ are the respective sample means of the *X*_1j_ and *X*_2k_ observations. Accordingly, ${\hat{\beta }}_{1D}={\hat{\beta }}_{11}-{\hat{\beta }}_{12}∼N\{\beta_{1D},\sigma^{2}(1/{SSX}_{1}+1/{SSX}_{2})\}$. On the other hand, ${\hat{\sigma }}^{2}=SSE/\nu$ is the usual unbiased estimator of *σ*^2^ where *SSE* is the error sum of squares and *ν* = *N*– 4. Moreover, *SSE*/*σ*^2^ ∼ *χ*^2^(*ν*), where *χ*^2^(*ν*) are chi-square distribution with *ν* degrees of freedom. To detect the difference between two slope coefficients in terms of H_0_: *β*_11_ = *β*_12_ versus H_1_: *β*_11_ ≠ *β*_12_, the test statistic has the form

$$T=\frac{{\hat{\beta }}_{1D}}{{\left\{{\hat{\sigma }}^{2}(1/{SSX}_{1}+1/{SSX}_{2})\right\}}^{1/2}}.$$ (4)

Under the null hypothesis H_0_: *β*_11_ = *β*_12_, the statistic has the distribution

T∼t(ν), (5)

where *t*(*ν*) is a *t* distribution with degrees of freedom *ν*. The null hypothesis is rejected at the significance level *α* if

$$|T|>t_{\nu ,\alpha /2},$$ (6)

where *t*_*ν*,*α*/2_ is the 100(1 – *α*/2) percentile of the distribution *t*(*ν*). Note that the inference setting is discussed here only from the perspective of a two-sided test. The same concepts may be readily extended to one-sided situations.

The statistical inferences about the heterogeneous slope effect are based on the conditional distribution of the continuous covariates. Therefore, the corresponding results would be specific to the particular values of the covariates. However, before conducting a research study, the actual values of covariates cannot be known in advance just as the primary responses. Under such circumstances, it is more suitable to employ the random or unconditional setup as explicated in Sampson [19]. The underlying similarities and differences between fixed and random models have also been thoroughly illuminated in Cramer and Appelbaum [20] and Raudenbush [21]. Despite the complexity associated with the unconditional properties of the test procedure, the tests of hypotheses and estimates of parameters remain the same under both conditional and unconditional frameworks. Hence, the usual rejection rule and critical value remain unchanged. The distinction between the two modeling approaches becomes important only when power and sample size calculations are to be made. Thus, it is vital to recognize the stochastic nature of the covariate variables and to evaluate the distribution of the test statistic over possible values of the covariates. In order to elucidate the critical notion of accommodating the distributional properties of the covariate variables, the continuous covariate variables {*X*_1*j*_, *j* = 1,…, *N*_1_} and {*X*_2*k*_, *k* = 1,…, *N*_2_} are assumed to have the independent normal distributions $N(\theta_{1},\tau_{1}^{2})$ and $N(\theta_{2},\tau_{2}^{2})$, respectively. It should be noted that the normality setting is commonly employed to provide a convenient framework for analytical derivation and theoretical discussion in interaction studies, for example, see Harwell [2], McClelland and Judd [22], O’Connor [23], and Shieh [14].

To help justify the contribution of current investigation, a brief review of the simple interaction model with two continuous covariates is presented here:

$$Y_{i}=\beta_{I}+X_{i}\beta_{X}+{Z_{i}\beta_{Z}+\;X_{i}Z_{i}\beta_{XZ}+\xi }_{i},$$ (7)

where *Y*_*i*_ is the value of the response variable *Y*, *X*_*i*_ and *Z*_*i*_ are the known constants of the continuous covariates *X* and *Z*, *ξ*_*i*_ are *iid N*(0,*ω*^2^) random errors for *i* = 1,…, *N*, and *β*_*I*_, *β*_*X*_, *β*_*Z*_, and *β*_*XZ*_ are unknown parameters. For the purpose of detecting the interaction effect in terms of the hypotheses H_0_: *β*_*XZ*_ = 0 versus H_1_: *β*_*XZ*_ ≠ 0, it is important to examine the distributional property for the least squares estimator ${\hat{\beta }}_{XZ}$ of *β*_*XZ*_:

$${\hat{\beta }}_{XZ}∼N\left(\beta_{XZ},V({\hat{\beta }}_{XZ})\right),$$ (8)

where $V({\hat{\beta }}_{XZ})=\omega^{2}M$, *M* is the (3, 3) element of ${\left(X_{C}^{\text{T}}X_{C}\right)}^{-1}$, where **X**_*C*_ = ${\left[X_{1}-\bar{X},\ldots ,X_{N}-\bar{X}\right]}^{\text{T}}$, $\bar{X}=\sum_{i=1}^{N}X_{i}/N$, and **X**_*i*_ = [*X*_*i*_, *Z*_*i*_, *X*_*i*_*Z*_*i*_]^T^ is the 3 × 1 column vector for values of covariates *X*_*i*_, *Z*_*i*_, and their cross product *X*_*i*_*Z*_*i*_ for *i* = 1,…, *N*. The corresponding test statistic *T*_*XZ*_ is of the form

$$T_{XZ}=\frac{{\hat{\beta }}_{XZ}}{{\left\{{\hat{\omega }}^{2}M\right\}}^{1/2}}$$ (9)

where ${\hat{\omega }}^{2}$ is the usual unbiased estimator of *ω*^2^. When the null hypothesis H_0_: *β*_*XZ*_ = 0 is true, the statistic *T*_*XZ*_ is distributed as *t*(*ν*), and H_0_ is rejected at the significance level *α* if |*T*_*XZ*_| > *t*_*ν*,*α*/2_. At first sight, all of the model structure, tested hypothesis, and decision rule are similar to the prescribed results given in Eqs 4–6 for detecting the treatment by covariate interaction. However, the two test statistics *T*_*XZ*_ and *T* have different forms and distribution properties under alternative hypothesis. Specifically, an alternative expression for the centered design matrix **X**_*C*_ is **X**_*C*_ = [**x**_*C*_, **z**_*C*_, **w**_*C*_] where **x**_*C*_, **z**_*C*_, and **w**_*C*_ are the three *N* × 1 column vectors of **X**_*C*_. Then, it can be shown that *M* = ${\left({w_{C}^{T}M}_{AC}w_{C}\right)}^{-1}$, $M_{AC}=I_{N}-X_{AC}{\left(X_{AC}^{T}X_{AC}\right)}^{-1}X_{AC}^{T}$ and **X**_*AC*_ = [**x**_*C*_, **z**_*C*_]. The complex expression of *M* generally does not have a simple analytic distribution even though the two covariate variables *X* and *Z* may have a bivariate normal distribution. It should be obvious that the product *XZ* of two normally distributed variables does not have a normal distribution. Hence, it is inaccessible to obtain a transparent nonnull distribution for the test statistic *T*_*XZ*_ under random or unconditional framework with a given joint distribution of *X* and *Z*. Instead, Shieh [14] adopted a large-sample viewpoint and considered the asymptotic distribution of *M*. The resulting nonnull distribution and associated power function of the statistic *T*_*XZ*_ are considerably more complicated than the explications presented later for the *T* test of treatment by covariate interactions. Consequently, the power and sample size calculations of Shieh [14] for detecting interactions between two continuous variables in multiple regression analysis are not applicable for assessing interactions between grouping and continuous variables within the context of ANCOVA. In the following, particular attention is given to develop useful and specialized statistical techniques for power and sample size computations in assessing the difference between two regression slopes.

In general, the statistic *T* has the nonnull distribution for the given values of *SSX*_1_ and *SSX*_2_:

$$T|[{SSX}_{1}{,SSX}_{2}]∼t(\nu ,\Delta ),$$ (10)

where *t*(*ν*,Δ) is a noncentral *t* distribution with degrees of freedom *ν* and noncentrality parameter

$$∆=\frac{\delta }{{\left(1/{SSX}_{1}+1/{SSX}_{2}\right)}^{1/2}},$$ (11)

where *δ* = *β*_1*D*_/*σ*. It follows from Johnson, Kotz, and Balakrishnan [24] that the first moment of a noncentral *t* distribution is *E*[*T*] = (*ν*/2)^1/2^Γ{(*ν*−1)/2}Δ/Γ{*ν*/2}, where Γ{∙} is the gamma function. Hence, an unbiased estimator of the effect size *δ* is

$${\hat{\delta }}_{UE}=\frac{{\left(1/{SSX}_{1}+1/{SSX}_{2}\right)}^{1/2}\Gamma \{\nu /2\}}{{\left(\nu /2\right)}^{1/2}\Gamma \{(\nu -1)/2\}}∙T=\frac{\Gamma \{\nu /2\}}{{\left(\nu /2\right)}^{1/2}\Gamma \{(\nu -1)/2\}}∙\frac{{\hat{\beta }}_{1D}}{\hat{\sigma }}.$$

To derive the nonnull distribution of *T*, an exact and sophisticated approach is to utilize the full distribution associated with *SSX*_1_ and *SSX*_2_. With the prescribed normal covariate assumptions, it can be readily established that *K*_1_ = ${SSX}_{1}/\tau_{1}^{2}\;∼\;\chi^{2}(\kappa_{1})$ and *K*_2_ = ${SSX}_{2}/\tau_{2}^{2}\;∼\;\chi^{2}(\kappa_{2})$ where *κ*_1_ = *N*_1_−1 and *κ*_2_ = *N*_2_−1. For ease of illustration, the two random variables of *K*_1_ and *K*_2_ are transformed to obtain *K* = *K*_1_ + *K*_2_ ~ *χ*^2^(*κ*) and *B* = *K*_1_/*K* ~ Beta{*κ*_1_/2, *κ*_2_/2} where Beta{*a*, *b*} is a beta distribution with degrees of freedom *a* and *b*. Note that the random variables *K* and *B* are independent. Under the prescribed stochastic considerations of *SSX*_1_ and *SSX*_2_ in terms of *K* and *B*, the *T* statistic has the following two-stage distribution

$$T|[K,\;B]∼t(\nu ,\Delta_{K}{}_{B}),\;K∼\chi^{2}(\kappa ),\;\text{and}\;B∼\mathrm{Beta}\{\kappa_{1}/2,\;\kappa_{2}/2\}.$$ (12)

where

$$\Delta_{KB}=\frac{\delta }{{\left\{[1/(B_{1}\tau_{1}^{2})+1/(B_{2}\tau_{2}^{2})]/K\right\}}^{1/2}},$$

*B*_1_ = *B*, and *B*_2_ = (1 –*B*). Hence, the resulting power function for comparing nonparallel regression lines is

$$\Psi_{KB}(\beta_{1D})=E_{K}E_{B}[P\{|t(\nu ,\Delta_{KB})|>t_{\nu ,\alpha /2}\}],$$ (13)

where the expectation *E*_*K*_[·] and *E*_*B*_[·] is taken with respect to the distribution of *K* and *B*, respectively.

Alternatively, a simple and naive method to obtain a unconditional distribution of *T* is to substitute the two sum of squares *SSX*_1_ and *SSX*_2_ in Δ with the corresponding expected values *E*[*SSX*_1_] = ${\kappa_{1}\tau }_{1}^{2}$ and *E*[*SSX*_2_] = ${\kappa_{2}\tau }_{2}^{2}$. Consequently, the distribution of *T* can be approximated by a noncentral *t* distribution as

$$T\dot{∼}t(\nu ,\Delta_{A}),$$ (14)

where

$$\Delta_{A}=\frac{\delta }{{\left\{[1/(b_{1}\tau_{1}^{2})+1/(b_{2}\tau_{2}^{2})]/\kappa \right\}}^{1/2}},$$

*b*_1_ = *κ*_1_/*κ*, *b*_2_ = *κ*_1_/*κ*, and *κ* = *κ*_1_ + *κ*_2_. The corresponding power function for the test for heterogeneity of regression slopes can be expressed as

$$\Psi_{A}(\beta_{1D})=P\{|t(\nu ,\Delta_{A})|>t_{\nu ,\alpha /2}\}.$$ (15)

On the other hand, Dupont and Plummer [13] presented a relatively more simplified power function for the test of difference between two regression slopes:

$$\Psi_{DP}(\beta_{1D})=P\{t(\nu )<\Delta_{DP}-t_{\nu ,\alpha /2}\}+P\{t(\nu )<{-\Delta }_{DP}-t_{\nu ,\alpha /2}\}.$$ (16)

where

$$\Delta_{DP}=\frac{\delta }{{\left\{[1/(p_{1}\tau_{1}^{2})+1/(p_{2}\tau_{2}^{2})]/N\right\}}^{1/2}},$$

*p*_1_ = *N*_1_/*N* and *p*_2_ = 1 –*p*_1_. Although the two noncentrality parameters Δ_*A*_ and Δ_*DP*_ are quite similar, especially when the sample size *N* is large, the two approximate power functions Ψ_*A*_ and Ψ_*DP*_ have a crucial difference. Note that the power function Ψ_*A*_ involves a noncentral *t* distribution *t*(*ν*,Δ_*A*_), whereas Ψ_*DP*_ is formulated through a shifted *t* distribution *t*(*ν*) + Δ_*DP*_). It is well known that if *Z* ~ *N*(0, 1) then *X* = (*Z* + μ) ∼ *N*(*μ*, 1) where μ is a constant. However, the result does not generalize to the case of *t* distribution, i.e., if *t* ~ *t*(*df*) then *Y* = (*t* + μ) does not follow a noncentral *t* distribution *t*(*df*, μ) with noncentrality parameter μ and degrees of freedom *df*. A random variable *Y* is said to have a noncentral *t* distribution *t*(*df*, μ) if and only if *Y* = (*Z* + μ)/(*W*/*df*)^1/2^ where *Z* ~ *N*(0, 1), *W* ~ *χ*^2^(*df*), and *Z* and *W* are independent. Essentially, Dupont and Plummer [13] extended the results under normal theory in Dupont and Plummer [25] to the case of noncentral *t* distributions in the comparison of two regression slopes. The resulting formulation suffers the absence of proper theoretical justification. Despite the computational appeal of the approximate power function Ψ_*DP*_, the prescribed analytic issue induces a fundamental question about its general adequacy as a reliable procedure.

It is essential to note that all the power functions Ψ_*DP*_, Ψ_*A*_ and Ψ_*KB*_ depend on the difference between two coefficients {*β*_11_, *β*_12_} and error variance *σ*^2^ through the standardized effect *δ*. Under the prescribed stochastic assumptions for the covariate variables, these power functions rely on the covariate variances {$\tau_{1}^{2}$, $\tau_{2}^{2}$} through the associated noncentrality parameter, but not the mean values of covariate variables {*θ*_1_, *θ*_2_}. Moreover, the approximate formulations of Ψ_*DP*_ and Ψ_*A*_ only involve the central *t* and noncentral *t* distributions, whereas the normal covariate distributions lead to the unique and more complex conditional property of Ψ_*KB*_ on the chi-square distribution and beta distribution. It can be shown that the noncentrality terms Δ_*DP*_, Δ_*A*_, and Δ_*KB*_ are asymptotically equivalent as sample size goes to infinity. Therefore, the three power functions Ψ_*DP*_, Ψ_*A*_, and Ψ_*KB*_ have the same large sample properties. Despite the close resemblance between the three power formulas, the corresponding behaviors for finite sample obviously differ. Their relative performance of power calculations will be appraised in the numerical investigations.

For planning research design, the power formulas can be employed to determine the sample sizes *N*_1_ and *N*_2_ needed to attain the specified power (1 – *β*) through a simple iterative search for the chosen significance level *α* and parameter settings. In practice, a research study requires adequate statistical power and sufficient sample size to detect scientifically credible effects. It is sensible that the corresponding power calculations and sample size determinations must be considered in the planning stage of a study. Consequently, it is of theoretical importance to evaluate the potential discrepancy between the three procedures in power and sample size calculations. In view of the wide variety of practical situations, the presumed normal covariate distribution merely provides a convenient and important situation. Evidently, the degree of robustness to nonnormal covariates for the resulting power and sample size procedures is also an essential issue and requires further sensitivity assessments.

## Simulation study

To justify the distinct advantage of the suggested exact approach and the potential deficiency of the approximate methods, numerical examinations of power and sample size calculations were conducted in two studies under a wide variety of model configurations. The first investigation focuses on the situations with normal covariate variables, whereas several notable scenarios of non-normal covariates are examined in the subsequent appraisal.

### Study I

For the purpose of explicating the critical discrepancy between the three power functions Ψ_*DP*_, Ψ_*A*_, and Ψ_*KB*_ in using covariate information, the two covariates *X*_1_ and *X*_2_ are assumed to have normal distributions with variances {$\tau_{1}^{2}$, $\tau_{2}^{2}$} = {1, 1} and {1, 3} for balanced design with *N*_1_ = *N*_2_ and {$\tau_{1}^{2}$, $\tau_{2}^{2}$} = {1, 1}, {1, 3}, and {3, 1} for unbalanced design with *N*_2_ = 3*N*_1_. As noted earlier, the power functions do not depend on the covariate means *θ*_1_ and *θ*_2_. Without loss of generality, they are set as *θ*_1_ = *θ*_2_ = 0. In addition, the selected configurations of treatment means and error variance are *β*_11_ = 0.50 and 0.75, *β*_12_ = 0, and *σ*^2^ = 1. Hence, the resulting standardized effect size has two different values *δ* = 0.50 and 0.75. Overall these considerations result in a total of 10 different combined arrangements. These combinations of different covariate structures, effect magnitudes, and sample size allocations were chosen to represent as much as possible the extent of characteristics that are likely to be encountered in actual applications.

**Fig 1:**
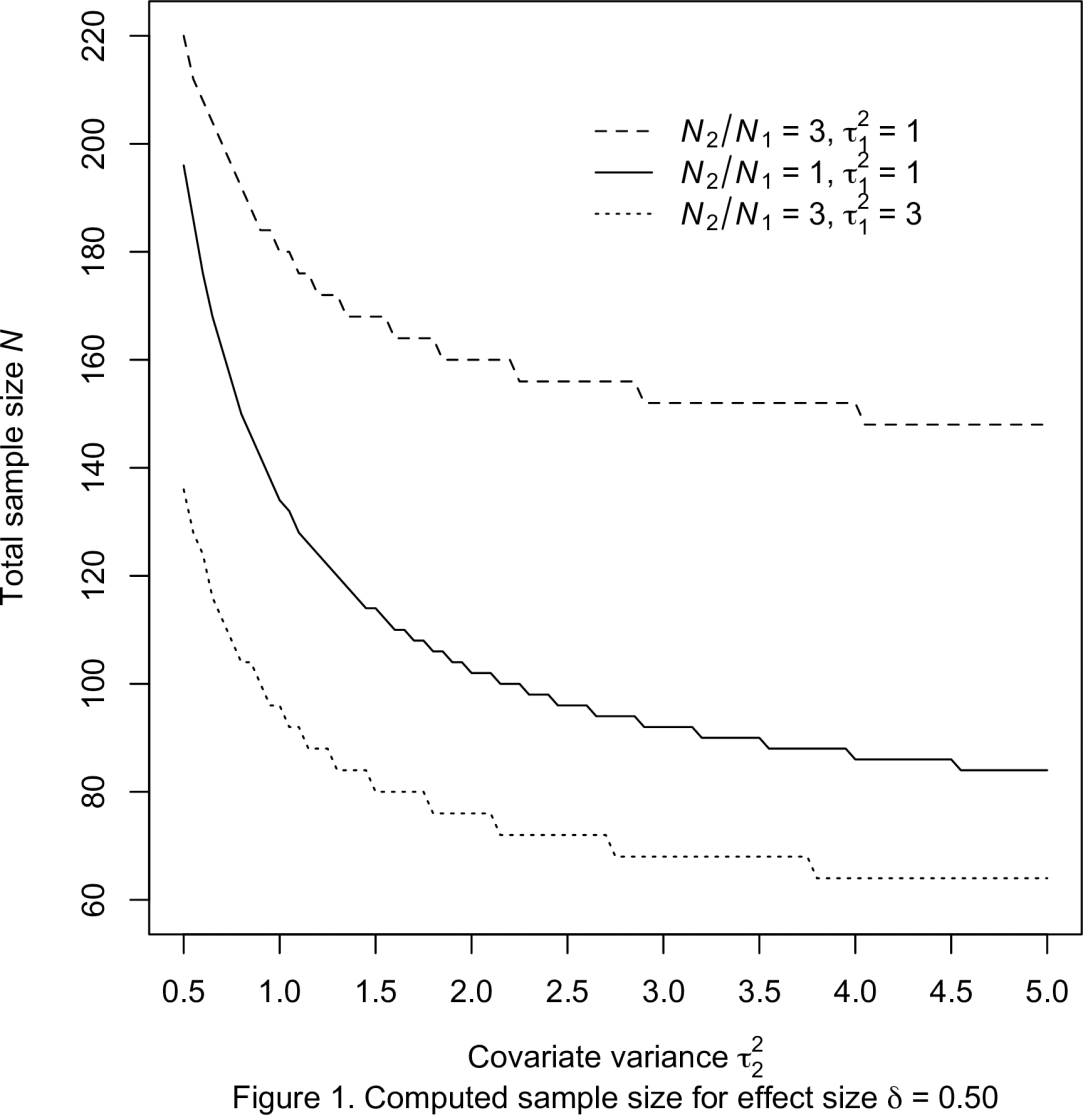
Computed sample size for effect size *δ* = 0.50.

**Fig 2:**
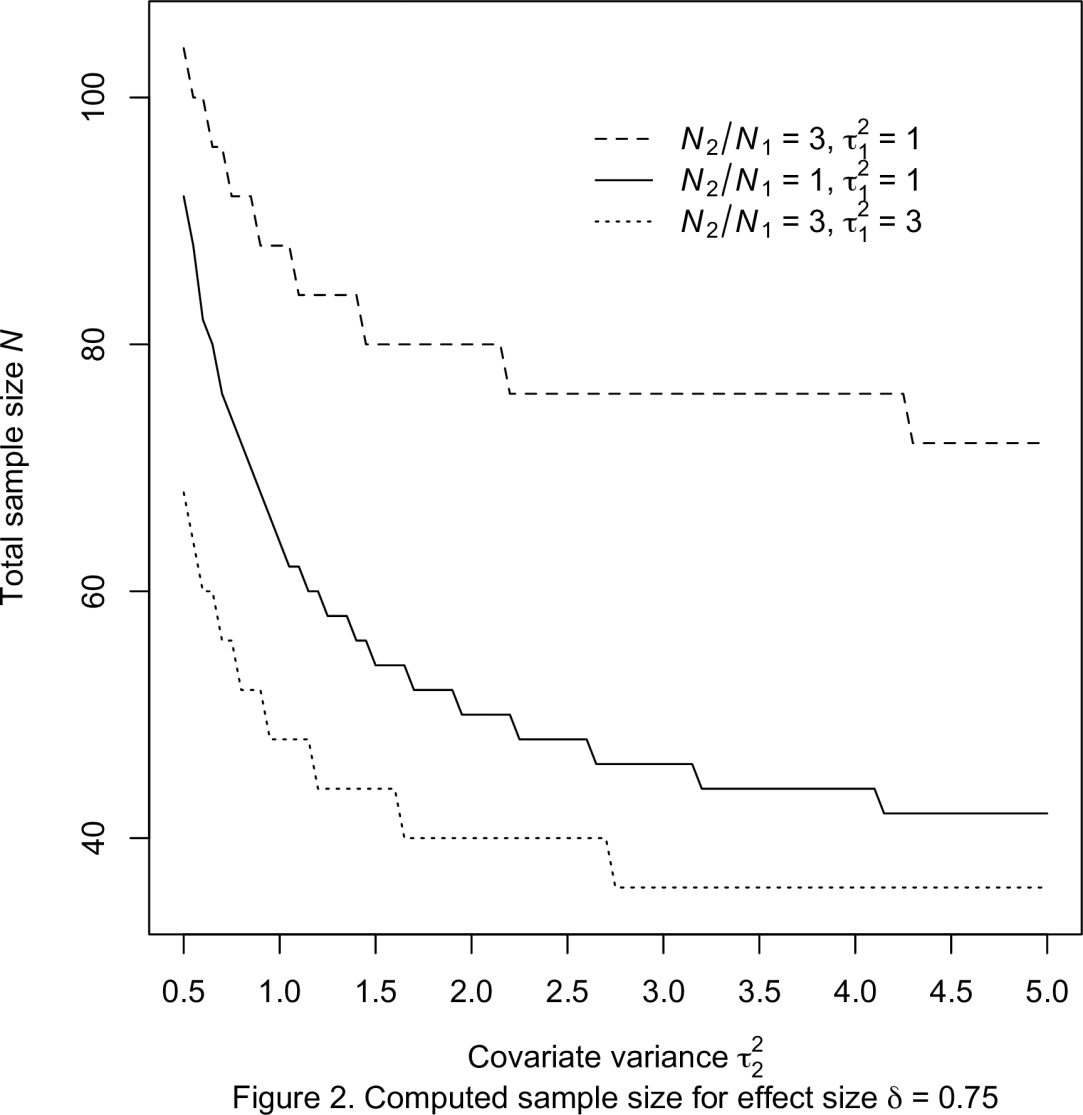
Computed sample size for effect size *δ* = 0.75.

With the prescribed specifications, the required sample sizes were computed for the three procedures with the chosen power value and significance level. Throughout this empirical investigation, the significance level and nominal power are fixed as *α* = 0.05 and 1 – *β* = 0.80, respectively. The computed sample sizes associate with the effect size *δ* = 0.50 and 0.75 are presented in Tables 1 and 2, respectively. For ease of illustration, the total sample sizes of the exact approach for *δ* = 0.50 and 0.75 are plotted in Figs 1 and 2, respectively.

**Table 1:** Computed sample size, estimated power, and simulated power when *δ* = 0.50, Type I error *α* = 0.05, and nominal power 1 – *β* = 0.80.

	Dupont and Plummer (1998)				Approximate method				Exact approach			
Covariate variance	Sample sizes	Estimated power	Simulated power	Error	Sample sizes	Estimated power	Simulated power	Error	Sample sizes	Estimated power	Simulated power	Error
{1, 1}	{64, 64}	0.8013	0.7773	0.0240	{65, 65}	0.8015	0.7859	0.0156	{67, 67}	0.8026	0.8000	0.0026
{1, 3}	{43, 43}	0.8011	0.7804	0.0207	{44, 44}	0.8015	0.7844	0.0171	{46, 46}	0.8037	0.8065	–0.0028
{1, 1}	{43, 129}	0.8059	0.7894	0.0165	{44, 132}	0.8076	0.7977	0.0099	{45, 135}	0.8033	0.8014	0.0019
{1, 3}	{36, 108}	0.8068	0.7831	0.0237	{37, 111}	0.8007	0.7943	0.0134	{38, 114}	0.8015	0.8011	0.0004
{3, 1}	{22, 66}	0.8103	0.7674	0.0429	{23, 69}	0.8165	0.7920	0.0245	{24, 72}	0.8122	0.8169	–0.0047

**Table 2:** Computed sample size, estimated power, and simulated power when *δ* = 0.75, Type I error *α* = 0.05, and nominal power 1 – *β* = 0.80.

	Dupont and Plummer (1998)				Approximate method				Exact approach			
Covariate variance	Sample sizes	Estimated power	Simulated power	Error	Sample sizes	Estimated power	Simulated power	Error	Sample sizes	Estimated power	Simulated power	Error
{1, 1}	{29, 29}	0.8008	0.7682	0.0326	{30, 30}	0.8014	0.7745	0.0269	{32, 32}	0.8045	0.8072	–0.0027
{1, 3}	{20, 20}	0.8068	0.7467	0.0601	{21, 21}	0.8080	0.7715	0.0365	{23, 23}	0.8135	0.8156	–0.0021
{1, 1}	{20, 60}	0.8180	0.7687	0.0493	{20, 60}	0.8016	0.7719	0.0297	{22, 66}	0.8125	0.8145	–0.0020
{1, 3}	{17, 51}	0.8236	0.7710	0.0526	{17, 51}	0.8020	0.7736	0.0284	{19, 57}	0.8124	0.8169	–0.0045
{3, 1}	{10, 30}	0.8068	0.7194	0.0874	{11, 33}	0.8211	0.7713	0.0498	{12, 36}	0.8126	0.8181	–0.0055

The graphs show that, for fixed values of sample size ratio *r* and covariate variance $\tau_{1}^{2}$, the total sample sizes *N* decrease with increasing covariance variance $\tau_{2}^{2}$. It is clear that the computed sample sizes in Table 1 are larger than those in Table 2 when all other characteristics are the same. More importantly, the results show that the calculated sample sizes of the exact approach differ from those of the two approximate procedures for all ten cases. The sample sizes of the approximate methods are relatively smaller than those of the exact approach. Also, the discrepancy are slightly larger for *δ* = 0.75 in Table 2 than those of *δ* = 0.50 in Table 1. In order to evaluate the accuracy of the power functions, the estimated power or computed power are also listed. Because of the underlying metric of integer sample sizes, the attained values are marginally larger than the nominal level for all three procedures.

Then, Monte Carlo simulation studies were performed to evaluate the accuracy of the sample size calculations. With the computed sample sizes, parameter configurations, and nominal power, estimates of the true power were computed via Monte Carlo simulation of 10,000 independent data sets. For each replicate, *N*_1_ and *N*_2_ covariate values were generated from the selected normal distributions. The resulting values of covariate variables in turn determined the mean responses for generating *N*_1_ and *N*_2_ normal outcomes with the designated ANCOVA designs. Next, the test statistic *T* was computed and the simulated power was the proportion of the 10,000 replicates whose test statistics |*T*| exceeded the corresponding critical value *t*_*ν*,0.025_. Therefore, the adequacy of the approximate and exact sample size procedures is determined by the error (= estimate power–simulated power) between the estimated power computed from analytic formulas and the simulated power of Monte Carlo study. The simulated power and error are also summarized in Tables 1 and 2 for all 10 design schemes.

It is noticeable from the results that there exists a close agreement between the estimated power and the simulated power for the proposed exact sample size procedure regardless of the model configurations. Specifically, all the incurred errors of the 10 designs are all within the small range of –0.0055 to 0.0026. In contrast, the estimated powers for the two approximate methods are consistently larger than the simulated powers for all 10 settings in Tables 1 and 2. In particular, the errors associated with Dupont and Plummer’s [13] procedure are {0.0240, 0.0207, 0.0165, 0.0237, 0.0429} and {0.0326, 0.0601, 0.0493, 0.0526, 0.0874} for *δ* = 0.50 and 0.75 in Tables 1 and 2, respectively. For the approximate method with power function Ψ_*A*_, the corresponding errors of the ten cases in Tables 1 and 2 are {0.0156, 0.0171, 0.0099, 0.0134, 0.0245} and {0.0269, 0.0365, 0.0297, 0.0284, 0.0498} for *δ* = 0.50 and 0.75, respectively. Although some of the differences are not substantial, it delineates a clear pattern that the accuracy of the approximate power functions deteriorates to some degree for smaller sample sizes, especially for the simple method of Dupont and Plummer [13]. Furthermore, the magnitudes of errors correspond to the direct-paring cases (when larger covariate variance is paired with larger sample size) are relative smaller than those of the inverse-pairing situations (when larger covariate variance is paired with smaller sample size). Note that the resulting errors of Dupont and Plummer’s [13] procedure associated with {$\tau_{1}^{2}$, $\tau_{2}^{2}$} = {1, 3} and {*N*_1_, *N*_2_} = {36, 108} and {17, 51} under direct-pairing are 0.0237 and 0.0526 in Tables 1 and 2, respectively. However, the counterparts of inverse-pairing setting with {$\tau_{1}^{2}$, $\tau_{2}^{2}$} = {3, 1} and {*N*_1_, *N*_2_} = {22, 66} and {10, 30} are much larger with 0.0429 and 0.0874 for *δ* = 0.50 and 0.75, respectively. These realizations imply that the magnitude of sample sizes plays an essential role in the performance of the approximate methods. More importantly, the adequacy of the approximate power formulas and sample size procedures varies with model configurations. In contrast, the numerical performance suggests that the exact methodology performs fairly well for the range of model specifications considered here.

### Study II

The described exact power function is obtained under the essential framework that the covariate variables have normal distributions. Instead of using the full features, the approximate power formula Ψ_*A*_ only relies on the partial information of second moments or variances of the covariates. At first sight, the simplified method may be more robust than the exact approach to the violation of normality assumption of the covariates. To further illuminate the sensitivity issues and profound implications of the two distinct techniques, power and sample size calculations were also conducted for the scenarios with non-normal covariates. Due to the undesired and inferior performance of Dupont and Plummer’s [13] technique, their method is not considered in this examination.

Specifically, the two covariates are assumed to have five different sets of distributions: Beta, Exponential, Gamma, Laplace, and Uniform. For ease of comparison, the designated distributions were constructed to have variances {$\tau_{1}^{2}$, $\tau_{2}^{2}$} = {1, 1} and {1, 3}. Moreover, only balanced designs were considered and the treatment means and error variance were fixed as *β*_11_ = 0.50, *β*_12_ = 0, and *σ*^2^ = 1. Hence, the required sample sizes and estimated powers associated with the exact procedure remain identical for the five different distributions. Unlike the previous study, the estimated powers and related evaluations of the approximate method were computed with the sample sizes determined by the exact approach. Table 3 summarizes the empirical results of the ten combined structures of covariate distribution and associated variance. In the case of Beta distribution, the actual two pairs of Beta covariates are *X*_1_ ~ Beta(2, 5)/*c*_1_ and *X*_2_ ~ Beta(2, 5)/*c*_1_, and *X*_1_ ~ Beta(2, 5)/*c*_1_ and *X*_2_ ~ Beta(2, 5)/*c*_2_ where *c*_1_ and *c*_2_ are selected such that the resulting variances are 1 and 3, respectively. On the other hand, the parameter specifications of the other four types of distribution can be found in Table 3. Similar to the numerical assessments in Study I, Table 3 presents the computed sample sizes, estimated powers, simulated powers, and associated errors of the two competing procedures.

**Table 3:** Computed sample size, estimated power, and simulated power when *δ* = 0.50, Type I error *α* = 0.05, and nominal power 1 – *β* = 0.80.

		Approximate method			Exact approach		
Covariate distributions	Sample sizes	Estimated power	Simulated power	Error	Estimated power	Simulated power	Error
Beta(2, 5)* and Beta(2, 5)*	{67, 67}	0.8135	0.7973	0.0162	0.8026	0.7973	0.0053
Beta(2, 5)* and Beta(2, 5)**	{46, 46}	0.8194	0.7960	0.0234	0.8037	0.7960	0.0077
Exponential(1) and Exponential(1)	{67, 67}	0.8135	0.7775	0.0360	0.8026	0.7775	0.0251
Exponential(1) and Exponential(3^1/2^)	{46, 46}	0.8194	0.7697	0.0497	0.8037	0.7697	0.0340
Gamma(2, 1/2^1/2^) and Gamma(2, 1/2^1/2^)	{67, 67}	0.8135	0.7905	0.0230	0.8026	0.7905	0.0121
Gamma(2, 1/2^1/2^) and Gamma(2, (3/2)^1/2^)	{46, 46}	0.8194	0.7830	0.0364	0.8037	0.7830	0.0207
Laplace(2^1/2^) and Laplace(2^1/2^)	{67, 67}	0.8135	0.7927	0.0208	0.8026	0.7927	0.0099
Laplace(2^1/2^) and Laplace((2/3)^1/2^)	{46, 46}	0.8194	0.7814	0.0380	0.8037	0.7814	0.0223
Uniform(–1/2, 1/2) and Uniform(–1/2, 1/2)	{67, 67}	0.8135	0.8115	0.0020	0.8026	0.8115	–0.0089
Uniform(–1/2, 1/2) and Uniform(–3, 3)	{46, 46}	0.8194	0.8095	0.0099	0.8037	0.8095	–0.0058

*Beta(2, 5) is scaled to have a variance 1

**Beta(2, 5) is scaled to have a variance 3.

A detailed inspection of the findings in Table 3 reveals that the performance of both the contending procedures is affected by the non-normal covariate settings, especially for the Exponential cases. However, it is important to note that the approximate technique incurs larger estimated powers and errors between estimated power and simulated power than the exact approach. The only exceptions occurred with the Uniform covariate distribution that the exact procedure does not have a clear advantage over the approximate method. Conceivably, the degree of robustness of the suggested exact technique presumably depends on the extent of how badly covariate distributions deviate from normality assumption. Nonetheless, these empirical evidences show that the exact procedure give acceptable results even for the non-normal covariates. In view of the potentially diverse treatment and covariate configurations of ANCOVA studies, it appears that the exact approach is relatively more consistent and accurate than the approximate method to be considered as a general tool.

## Results

The implementation of the suggested power and sample size calculations involves specialized programs not currently available in prevailing statistical packages. To exemplify the computational aspects of the developed algorithms for design planning, the numerical demonstration of evaluating two treatments for gingivitis in Fleiss [3, Section 7.3] is reexamined here. The data consists of measurements of patients before and after treatment on a modification of the Loe and Silness [26] index of gingivitis. A higher value indicates a more severe level of gingivitis. Accordingly, the response variable of ANCOVA is the post-treatment measurement with the pretreatment value serving as the covariate. It should be note that the illustration in Fleiss [3] does not address the power and sample size issues. Moreover, the emphasis of this numerical demonstration is on the typical research scenario most frequently encountered in the planning stage of an ANCOVA study.

Due to the prospective nature of advance research planning, the general guidelines suggest that typical sources like published finding or expert opinion can offer plausible and reasonable planning values for the model characteristics, such as treatment effects, variance component, and covariate properties. To explicate the essential processes, the prescribed data of comparing two treatments of gingivitis is employed to provide planning values of the model parameters and covariate configurations for related gingivitis studies. Specifically, the summary statistics yield the designated treatment effects and variance component: *β*_11_ = 0.8502, *β*_12_ = 0.4008, and *σ*^2^ = 0.04. In addition, the covariate variances are obtained from the reported pretreatment values as $\tau_{1}^{2}$ = 0.0646 and $\tau_{2}^{2}$ = 0.0526. With the sample sizes of {*N*_1_, *N*_2_} = {74, 64} and significance level *α* = 0.05, the achieved power can be readily computed with the supplemental programs (Programs A and C). The result shows that the achieved power of the particular unbalanced design is Ψ_*KB*_ = 0.8650 which falls between the two fairly common levels of 0.80 and 0.90. Therefore, the power calculation suggests that the designated configurations warrant a decent chance of detecting the slope difference between two treatment groups.

Alternatively, under the notion of a balanced design, the presented algorithms (Programs B and D) reveal that the equal sample sizes of {*N*_1_, *N*_2_} = {69, 69} yield the power of 0.8694. It is interesting to note that, although the two sample size schemes {74, 64} and {69, 69} have the identical total sample size 138, the balanced design has a slightly advantage over the unbalanced structure in power performance. For an illustration of sample size determination for planning balanced study, detailed computations show that the balanced sample sizes of {*N*_1_, *N*_2_} = {58, 58} and {77, 77} are needed to achieve the target powers of 0.80 and 0.90, respectively. It is noted above, because of the sample sizes need to be integer values in practice, that the attained power is marginally greater than the nominal power level. Here, the corresponding actual powers of the two sample size designs are 0.8043 and 0.9038, respectively. These vital configurations are incorporated in the user specifications of the SAS/IML [13] and R [14] programs presented in the supplemental files. With the prescribed explications, users can easily identify the statements containing the exemplifying values in the computer code and then modify the program to accommodate their own model specifications.

## Conclusions and discussion

Within the context of ANCOVA, an underlying assumption is the parallelism of the regression lines associating the criterion variable with the covariate. It has been emphasized that the homogeneity of covariate regression slopes is the most important statistical assumption in ANCOVA. However, there are theoretical reasons and empirical evidences to document nonparallel phenomenon of regression lines across many scientific fields. Although the test of the hypothesis of parallel regression lines is a simple and straightforward procedure, the corresponding analytic derivations and computational algorithms of power and sample size determinations have not been examined in the literature. Conceivably, the corresponding power analysis and sample size determination must also be considered before it can be adopted as a general methodology in practice. To facilitate proper use and implication of traditional ANCOVA and extended alternatives, this article presents both pedagogical explication and numerical appraisal of power and sample size procedures for the detection of heterogeneity between two covariate regression coefficients. Despite the simplicity, this scenario embodies all the essential notion and critical feature of ANCOVA that can be useful in undertaking similar considerations for the more involved multi-group situations.

The existing method of Dupont and Plummer (1998) seems to provide a simple solution and maintains reasonable accuracy for some model configurations. However, no research to date has properly examined its properties both analytically and empirically. The presented analytic explication and empirical results showed that the approximate formula of Dupont and Plummer [13] does not guarantee to give accurate power and sample size calculations. The proposed exact approach has the distinct feature of accommodating the full distributional properties of normal covariates whereas the simplified approximate methods only utilize the partial information of covariate variances. It is important to note that although Glueck and Muller [27] and Shieh [28] considered the problem of adjusting power for random covariates in multivariate linear models, their model formulations do not cover the interaction effects between treatment groups and continuous covariates. Hence, the corresponding power and sample size procedures do not applied to the detection of slope heterogeneity considered here. Moreover, due to the complexity of multivariate settings, only moments of the covariate variables are employed in the power formulas presented in Glueck and Muller [27] and Shieh [28]. Consequently, their methods do not take into account the full distributional features of covariate variables. In view of the overall accuracy and robustness, the exact approach is recommended over the approximate methods as a reliable tool in practical applications. The supporting SAS/IML [15] and R [16] computer algorithms will yield accurate power calculations and sample size determinations provided that all the required information is properly specified.

## Supporting information

S1 File SAS programs. (DOCX)

S2 File R programs. (DOCX)
