# Supplementary material for: On tests of treatment-covariate interactions: An illustration of appropriate power and sample size calculations
Source: PLoS One. 2017 May 17;12(5):e0177682. doi: 10.1371/journal.pone.0177682 (PMC5435249; doi:10.1371/journal.pone.0177682)
Supplement: S1 File — (DOCX) [file pone.0177682.s001.docx]

S1 File-SAS programs

Program A

SAS/IML program for computing the power for the tests of heterogeneity between two regression slopes

PROC IML;

*USER SPECIFICATION PORTION;

*DESIGNATED ALPHA;ALPHA=0.05;

*TREAMENT MEANS;BETA1=0.8502;BETA2=0.4008;

*ERROR VARIANCE;SIGSQ=0.04;

*GROUP SIZES;N1=74;N2=64;

*COVARIATE VARIANCES;TAUSQ1=0.0646;TAUSQ2=0.0526;

*END OF USER SPECIFICATION PORTION;

BETAD=BETA1-BETA2;SIGMA=SQRT(SIGSQ);DEL=BETAD/SIGMA;

NUMINT=50;L=NUMINT+1;DD=1E-6;

COEVEC=({1}||REPEAT({4 2},1,NUMINT/2-1)||{4 1})`;

BL=DD;BU=1-DD;INTB=(BU-BL)/NUMINT;BVEC=BL+(INTB#(0:NUMINT))`;

START KBPOWER;

DF=N1+N2-4;TCRIT=TINV(1-ALPHA/2,DF);

DFK1=N1-1;DFK2=N2-1;DFK=N1+N2-2;

DFB1=DFK1/2;DFB2=DFK2/2;

WBPDF=(INTB/3)#COEVEC#PDF('BETA',BVEC,DFB1,DFB2);

CL=DD;CU=QUANTILE('CHISQ',1-DD,DFK);

INTC=(CU-CL)/NUMINT;CVEC=CL+(INTC#(0:NUMINT))`;

WCPDF=(INTC/3)#COEVEC#PDF('CHISQ',CVEC,DFK);

QUAN=J(L,1,0);

DO I=1 TO L;B1=BVEC[I,1];B2=1-B1;

DELTAKBVEC=DEL/SQRT((1/(B1#TAUSQ1)+1/(B2#TAUSQ2))/CVEC);

QUAN[I,1]=WCPDF`*(CDF('T',-TCRIT,DF,DELTAKBVEC)+SDF('T',TCRIT,DF,DELTAKBVEC));

END;KBPOWER=WBPDF`*QUAN;

FINISH;

RUN KBPOWER;PRINT N1 N2 KBPOWER[FORMAT=8.4];

QUIT;

Program B

SAS/IML program for computing the sample size for the tests of heterogeneity between two regression slopes

PROC IML;

*USER SPECIFICATION PORTION;

*DESIGNATED ALPHA;ALPHA=0.05;

*NOMINAL POWER;POWER=0.9;

*TREAMENT MEANS;BETA1=0.8502;BETA2=0.4008;

*ERROR VARIANCE;SIGSQ=0.04;

*GROUP SIZE RATIO (=> 1);RN21=1;

*COVARIATE VARIANCES;TAUSQ1=0.0646;TAUSQ2=0.0526;

*END OF USER SPECIFICATION PORTION;

BETAD=BETA1-BETA2;SIGMA=SQRT(SIGSQ);DEL=BETAD/SIGMA;

NUMINT=50;L=NUMINT+1;DD=1E-6;

COEVEC=({1}||REPEAT({4 2},1,NUMINT/2-1)||{4 1})`;

BL=DD;BU=1-DD;INTB=(BU-BL)/NUMINT;BVEC=BL+(INTB#(0:NUMINT))`;

START KBPOWER;

DF=N1+N2-4;TCRIT=TINV(1-ALPHA/2,DF);

DFK1=N1-1;DFK2=N2-1;DFK=N1+N2-2;

DFB1=DFK1/2;DFB2=DFK2/2;

WBPDF=(INTB/3)#COEVEC#PDF('BETA',BVEC,DFB1,DFB2);

CL=DD;CU=QUANTILE('CHISQ',1-DD,DFK);

INTC=(CU-CL)/NUMINT;CVEC=CL+(INTC#(0:NUMINT))`;

WCPDF=(INTC/3)#COEVEC#PDF('CHISQ',CVEC,DFK);

QUAN=J(L,1,0);

DO I=1 TO L;B1=BVEC[I,1];B2=1-B1;

DELTAKBVEC=DEL/SQRT((1/(B1#TAUSQ1)+1/(B2#TAUSQ2))/CVEC);

QUAN[I,1]=WCPDF`*(CDF('T',-TCRIT,DF,DELTAKBVEC)+SDF('T',TCRIT,DF,DELTAKBVEC));

END;KBPOWER=WBPDF`*QUAN;

FINISH;

N1=9;LOOP=0;

DO UNTIL (KBPOWER>POWER | LOOP>1000);

N1=N1+1;N2=N1#RN21;LOOP=LOOP+1;

RUN KBPOWER;END;KBN1=N1;KBN2=N2;

PRINT KBN1 KBN2 KBPOWER[FORMAT=8.4];

QUIT;
