# Supplementary material for: On tests of treatment-covariate interactions: An illustration of appropriate power and sample size calculations
Source: PLoS One. 2017 May 17;12(5):e0177682. doi: 10.1371/journal.pone.0177682 (PMC5435249; doi:10.1371/journal.pone.0177682)
Supplement: S2 File — (DOCX) [file pone.0177682.s002.docx]

S2 File-R programs

Program C

R program for computing the power for the tests of heterogeneity between two regression slopes

function () {

#USER SPECIFICATION PORTION

alpha<-0.05 #DESIGNATED ALPHA

beta1<-0.8502 #TREATMENT MEANS

beta2<-0.4008

sigsq<-0.04 #ERROR VARIANCE

n1<-74 #GROUP SIZES

n2<-64

tausq1<-0.0646 #COVARIATE VARIANCES

tausq2<-0.0526

#END OF SPECIFICATION

betad<-beta1-beta2

sigma<-sqrt(sigsq)

del<-betad/sigma

numint<-50

l<-numint+1

dd<-1e-6

coevec<-c(1,rep(c(4,2),numint/2-1),4,1)

bl<-dd

bu<-1-dd

intb<-(bu-bl)/numint

bvec<-bl+intb*(0:numint)

kbpowerf<-function(){

df<-n1+n2-4

tcrit<-qt(1-alpha/2,df)

dfk1<-n1-1

dfk2<-n2-1

dfk<-n1+n2-2

dfb1<-dfk1/2

dfb2<-dfk2/2

wbpdf<-(intb/3)*coevec*dbeta(bvec,dfb1,dfb2)

cl<-dd

cu<-qchisq(1-dd,dfk)

intc<-(cu-cl)/numint

cvec<-cl+intc*(0:numint)

wcpdf<-(intc/3)*coevec*dchisq(cvec,dfk)

quan<-rep(0,l)

for (i in seq(l)) {

b1<-bvec[i]

b2<-1-b1

deltakbvec<-del/sqrt((1/(b1*tausq1)+1/(b2*tausq2))/cvec)

quan[i]<-sum(wcpdf*(pt(-tcrit,df,deltakbvec)+

pt(tcrit,df,deltakbvec,lower.tail=FALSE)))

}

kbpower<-sum(wbpdf*quan)

}

kbpower<-kbpowerf()

print("n1,n2,kbpower")

print(c(n1,n2,kbpower),digits=4)

}

Program D

R program for computing the sample size for the tests of heterogeneity between two regression slopes

function () {

#USER SPECIFICATION PORTION

alpha<-0.05 #DESIGNATED ALPHA

power<-0.8 #NOMINAL POWER

beta1<-0.8502 #TREATMENT MEANS

beta2<-0.4008

sigsq<-0.04 #ERROR VARIANCE

rn21<-1 #GROUP SIZE RATIO

tausq1<-0.0646 #COVARIATE VARIANCES

tausq2<-0.0526

#END OF SPECIFICATION

betad<-beta1-beta2

sigma<-sqrt(sigsq)

del<-betad/sigma

numint<-50

l<-numint+1

dd<-1e-6

coevec<-c(1,rep(c(4,2),numint/2-1),4,1)

bl<-dd

bu<-1-dd

intb<-(bu-bl)/numint

bvec<-bl+intb*(0:numint)

kbpowerf<-function(){

df<-n1+n2-4

tcrit<-qt(1-alpha/2,df)

dfk1<-n1-1

dfk2<-n2-1

dfk<-n1+n2-2

dfb1<-dfk1/2

dfb2<-dfk2/2

wbpdf<-(intb/3)*coevec*dbeta(bvec,dfb1,dfb2)

cl<-dd

cu<-qchisq(1-dd,dfk)

intc<-(cu-cl)/numint

cvec<-cl+intc*(0:numint)

wcpdf<-(intc/3)*coevec*dchisq(cvec,dfk)

quan<-rep(0,l)

for (i in seq(l)) {

b1<-bvec[i]

b2<-1-b1

deltakbvec<-del/sqrt((1/(b1*tausq1)+1/(b2*tausq2))/cvec)

quan[i]<-sum(wcpdf*(pt(-tcrit,df,deltakbvec)+

pt(tcrit,df,deltakbvec,lower.tail=FALSE)))

}

kbpower<-sum(wbpdf*quan)

}

n1<-9

loop<-0

kbpower<-0

while(kbpower<power & loop<1000){

n1<-n1+1

n2<-n1*rn21

loop<-loop+1

kbpower<-kbpowerf()

}

kbn1<-n1

kbn2<-n2

print("kbn1,kbn2,kbpower")

print(c(kbn1,kbn2,kbpower),digits=4)

}
